# AIDS-related lymphoma at the University of calabar teaching hospital (Nigeria): a seven year review

**DOI:** 10.1186/1750-9378-7-S1-P1

**Published:** 2012-04-19

**Authors:** Marcus A Inyama, IA Ibanga, Godwin Ebughe, I Bassey, Ima-Obong Ekanem, ME Asuquo

**Affiliations:** 1 Department of Haematology and Blood Transfusion, University of Calabar Teaching Hospital, Calabar, Nigeria; 2 Department of Pathology, University of Calabar Teaching Hospital, Calabar, Nigeria; 3 Department of Surgery, University of Calabar Teaching Hospital, Calabar, Nigeria

## Background

With the use of HAART and effective treatment, prolongation of life of persons living with HIV, there has been a steady increase in cases of AIDS-related lymphoma (ARL). The number keeps increasing. The complexity of managing patients with ARL in a resource limited setting needs to be evaluated in view of the diagnostic and managerial challenges.

## Objectives

To determine the prevalence of ARL in our hospital. To evaluate the outcome of patients management.

## Subjects and methods

Hospital records of patients with tissue diagnosis of lymphoma from January 2005-June 2011 were examined. Those with retroviral positive results were further followed up to treatment centre at the Presidential Emergency Programme for AIDS Relief (PEPFAR) clinic. Records were also reviewed at the Calabar Cancer Registry.

## Results

Fifty-four patients with lymphoma were seen within the period (2005-2011).Non-Hodgkin’s lymphoma (NHL) was the most frequent 35(63%) Hodgkin’s lymphoma (HL) 8(18.2%), Burkitt’s lymphoma(BL) 7(15.9%) and nasopharyngeal lymphoma(NL) 1(2.3%). ARL was 12(18.2%) with NHL, HL, and NL contributing 9(62%), 2(25%), 1(12.5%) respectively. Mortality was significantly higher in the ARL than in the non-ARL group.

## Conclusions

ARL is not a rarity in our environment. A survey of all the HIV treatment centres will reveal a larger statistics. A greater understanding of the biology of this complex is needed. Training of `all care providers to effectively manage the disease is highly recommended. The cost of drugs for the treatment of the lymphoma is prohibitive to most of the indigent patients who present to our centre.

